# Ethical biopsy

**DOI:** 10.1002/ncp.70011

**Published:** 2025-08-22

**Authors:** Albert Barrocas, Alison Evans

**Affiliations:** ^1^ Tulane University School of Medicine New Orleans Louisiana USA; ^2^ Amerita Specialty Infusion Duluth Georgia USA

**Keywords:** administration, enteral nutrition, life cycle, long‐term care, medical/nutrition ethics, nutrition support teams, oncology, parenteral nutrition, public policy, reimbursement

## Abstract

Artificially administered nutrition and hydration (AANH) often trigger complex decision‐making that intersects medical technology, ethical practice, and legal responsibility. Inspired by ASPEN's 50th anniversary clinical ethics discussions, this article introduces the concept of an “ethical biopsy,” a structured ethical inquiry process modeled after the diagnostic medical biopsy. By aligning medical and ethical criteria, this framework equips clinicians, nutrition support teams, and ethics committees with a compassionate and patient‐centered method for resolving dilemmas. Just as a biopsy is invasive but necessary, confronting ethical dilemmas is a crucial but often uncomfortable process for reaching patient‐centered decisions surrounding AANH, particularly at the end of life. A case report is presented of a patient with untreatable, advanced cancer who wishes to have parenteral nutrition with the goal of living longer and spending more time with her young children, although her medical team disagrees because of concerns for risk of infection. Components of an ethical biopsy are presented that can be used to resolve such an ethical dilemma.

## INTRODUCTION

During ASPEN's 50th anniversary celebration at the 2025 Science and Practice Conference, the International Clinical Ethics Section hosted a session on artificially administered nutrition and hydration (AANH) and palliative care. Audience members and faculty shared challenges in navigating these ethically complex decisions, often hindered by clinical ambiguity, ethical tension, financial constraints, and/or regulatory confusion.

This prompted a moment of reflection, a “cerebral singultus,” raising the question in the lead author's mind: Could ethical dilemmas be approached diagnostically, akin to a suspicious mass of unknown origin? Might we consider an “ethical biopsy” as a tool for evaluation and resolution?

The purpose of this article is to explore the feasibility of enhancing the success of professional and patient interactions in resolving dilemmas that arise in nutrition care. This article explores that approach, drawing from the ethical dimension of The Troubling Trichotomy (T3), the intersection of what can be done technologically, what should be done ethically, and what must be done legally.^1^ We propose a structured framework to facilitate resolution of ethical dilemmas and close practice gaps in the application of ethical principles in clinical practice.

## BACKGROUND

T3 has been described previously. Its application at the bedside (clinical dimension) and the community at large (ethical dimension) has the potential for ameliorating dilemmas covering all three.^1^ Dilemmas that arise at the bedside, varying from intrafamilial divergence of opinions regarding the forgoing of ANNH to making the case for palliative care by a reluctant physician, necessitate a framework for resolution. Clinical ethics education is often lacking in undergraduate and graduate healthcare training. Our proposal presents an ethical analog to clinical decision‐making, the ethical biopsy.

### Conceptual framework: The ethical biopsy

As envisioned, the ethical biopsy can provide a framework for applying ethical principles to reach informed, compassionate choices and consent when dealing with a variety of dilemmas emanating from ethical, administrative, financial, and legal perspectives, particularly when considering nutrition support therapies at end of life (EOL). By framing it in the context of a clinical analogy, the proposed framework has the potential for broader acceptance and success when used with a patient‐centered process that incorporates empathy. In clinical medicine, a biopsy aids diagnosis by analyzing tissue. An ethical biopsy examines situations in which ethical ambiguity, uncertainty, and knowledge gaps obstruct decision‐making. It can provide moral clarity through a coordinated, compassionate, and inclusive, transdisciplinary process.

Whether performed before, during, or at end of hospitalization, the ethical biopsy may prove to be a useful tool to ensure a patient‐centered approach to resolving ethical dilemmas. Application of an ethical biopsy after discharge can also lead to improved patient‐centered care not achieved at the time of hospital discharge.

This approach ensures that ethical principles and concerns, especially surrounding AANH, are not relegated to philosophical abstraction. The individual's autonomy and self‐determination are not disregarded because of healthcare professionals' opinions or beliefs and are considered with clinical rigor, transdisciplinary input, and empathetic dialogue (Table 1).

**Table 1:** Comparison of a medical and ethical biopsy regarding AANH with application to the case example.

Criteria	Medical Biopsy	Ethical Biopsy	Application to example case
**Rationale**	Seek diagnosis	Seek conflict resolution^3^, ^8^	✓ Deliberations that consider all issues and stakeholders ✓ Shared decision‐making via patient centered process^6^ ✓ Analyze benefits versus burdens/risks, medical indication ✓ Be open to sacred moments during the process ✓ Apply evidenced based medicine (EBM) and clinical expertise^6^ ✓ Apply an empathetic presence, active listening, and therapeutic silence ✓ Respect values, cultural and religious diversity, health equity^7^
**People**	Surgeon/Interventionalist Pathologist	Patient, family, significant others^7^ Physician, nurse, dietitian, pharmacist^7^ Clergy, ethicist Social worker/DC planner Other participants may also be appropriate	✓ Ethics committee, risk management, nutrition support team‐transdisciplinary approach ✓ Administrators, policy makers and legislators ✓ Payers/reimbursement, patient advocacy ✓ Consultation with the transdisciplinary team ✓ Consultation with case management and “receiving” outpatient teams (hospice, home health agency, home infusion pharmacy)
**Prerequisites for medical team**	Appropriate medical facility Training/experience of surgeon/interventionist, pathologist	Clinicians (preferably with ethical competencies^2^) and comfortable with: T3‐Clinical/ethical dimensions^6^ Principlism^1^, ^2^, ^3^, ^4^, ^5^, ^6^ ○ Autonomy ○ Beneficence ○ Non‐maleficence ○ Justice Ethical competencies^2^ Soft and behavioral skills Therapeutic silence Empathetic presence Pt‐centered cue‐based discussions	✓ Autonomy: honoring patient wishes and quality of life (QOL) goals ✓ Beneficence: treatment in the best interest of the patient ✓ Nonmaleficence: not harming an individual with a medical treatment or forgoing a beneficial treatment that could have been provided ✓ Justice ✓ Use of soft and behavioral skills ✓ Respect values, cultural and religious diversity, health equity^7^ ✓ Empathetic and compassionate language, therapeutic silence ✓ Cue based discussions and open‐ended questions ✓ Reflective practice, moral character ✓ Patient understands benefits vs. risks/burdens of treatment as well as consequences of forgoing treatment^3^ ✓ Education using the teach back method^3^ ✓ Compassionate language and empathetic presence
**Post‐Diagnosis**	Therapeutic‐Palliative options Treatment plan	Pt‐centered treatment plan Time limited trials^2^ Empathetic caring Tailored healthcare^3^ Payer engagement with respect and dignity^4^	✓ Tailored healthcare^3^ ✓ Compassionate, dignified, and respectful care

Abbreviations: DC, discharge; EBM, evidenced‐based medicine; HIPAA, Health Insurance Portability and Accountability Act of 1996; QOL, quality of life.

### Clinical application

The concept of the ethical biopsy emanated as a result of the International Clinical Ethics Section presentation. To illustrate its utility, a case report, discussed during the 2025 ASPEN Science and Practice Conference, is included with an ethical biopsy “checklist.” The ethical biopsy is applied to the case example in retrospect.

### Case example

Let us now enter the journey of a female middle aged patient with advanced, metastatic ovarian cancer, small bowel obstruction (SBO), significant peritoneal implants, and malignant ascites. The patient had severe malnutrition with a weight loss of 8% body weight the past 6 weeks and a total weight loss of 17%. She had not tolerated significant oral intake for >2 weeks. The patient was hospitalized, and palliative care was consulted. The patient was no longer a candidate for surgery or chemotherapy. A percutaneous transesophageal gastrostomy (TPEG) was placed for decompression and comfort. Hospice was offered. The patient and family were overwhelmed. However, the patient was of sound mind with decision‐making capacity and able to discern the dire implications of her advanced cancer. The patient's expressed wishes and goals of care included to live longer and to be at home for as long as possible with her family and young children who were aged 7 and 10 years. The patient requested parenteral nutrition (PN) to attempt to prolong her life. PN was not recommended by her medical team because of concerns of associated infection risk. Home hospice was offered, but the patient declined because only intravenous fluids (IVFs) were offered. PN was not offered by hospice because of its misalignment with hospice philosophy and its cost and associated complexity. Ethical tensions ensued. The patient was discharged without PN, hospice, or an ethical consultation and transitioned home with TPEG via continuous gastric decompression, clear liquids as tolerated, and IVF as needed. The patient was advised to follow up with her oncologist.

In retrospect, the application of the ethical biopsy at the time of the patient's initial request for PN would have resulted in earlier and better outcomes, particularly in the realm of delivering timely quality of care. Clinical management lacked a structured approach in navigating ethical complexities in considering PN at EOL. Integrating an ethical framework, or ethical biopsy, can help to identify and bridge gaps in ethical knowledge, reasoning, and application in clinical decision‐making. Insight gained from the ethical biopsy framework aids in uncovering systemic and/or implicit biases, as well as embedding ethical principles into everyday decision‐making. Through the ethical biopsy lens, clarity is gained and care aligns with ethical principles and patient‐centered goals (Table 1). Key aspects of the ethical biopsy in this case would have included the following:

(1) Consult with the ethics team, palliative care, primary oncologist, nutrition support team/clinician, spiritual health, forming the transprofessional team, to address all issues with a focus on a patient‐centered process via shared decision‐making to reach final, informed decisions regarding PN at EOL. Use compassionate language, empathetic presence, and therapeutic silence when discussing with the patient and family.
(2) Apply evidenced‐based medicine and discern the medical indication for PN, which was SBO/TPEG with inability to absorb oral nutrients in the setting of advanced cancer and severe malnutrition. Analyze the benefits vs risks/burdens of PN in EOL.
(3) Apply the four core ethical principles throughout the process:
  - Autonomy: Patient expressed her wishes and goals of care to have PN to help lengthen her life and have more time with her children.
  - Beneficence (in the best interest of the patient): Provide a medical treatment in the best interest of the patient. In this case, there was a medical indication for PN, and without PN, the patient would likely die from starvation vs the disease process itself. Despite the diagnosis, this young patient continued to experience moments of joy, dignity, and connection: clear markers of a meaningful quality of life.
  - Nonmaleficence (do no harm): Address through deliberations not harming an individual with a medical treatment or forgoing a beneficial treatment that could have been provided. When considering a medical treatment, that is PN, reduce complications by central line bundles, teaching/addressing daily/weekly catheter care and PN monitoring to mitigate associated complications.
  - Justice: Applying fair and equitable treatment for all patients, avoiding clinician biases.
(4) Educate the patient and family regarding PN applying the teach‐back method. Patient understands benefits vs risks/burdens of treatment as well as consequences of forgoing treatment.
(5) Arrive at final, informed decisions and medical interventions that provide tailored healthcare with respect, dignity, and compassion that are ethically sound. Enter sacred moments in which vulnerability, trust, and humanity converge.

### Outcomes of the case example

The patient followed up with her oncologist 1 week postdischarge. Since discharge, the patient received multiple doses of IVFs, paracentesis, and a blood transfusion. The patient again requested PN to have more time with her two young children and potentially to pursue other cancer treatments. The patient relayed to her oncologist, “I don't want to die yet.” A home‐initiated trial of PN was ordered by the oncologist.

The home infusion nutrition support dietitian met with the outpatient clinical team, patient, and her husband in clinic. Despite her emaciation, the patient had a smile on her face. Her wishes and goals of care were discussed intensively, and the patient reiterated wanting to be at home and “live” to share her life with her children for as long as possible. Benefits vs burdens and risks of PN, as well as refeeding risk, were discussed. A “show and tell” of equipment and the home‐initiated PN process was provided. A time‐limited trial and potential need to “hold and reevaluate” PN in the setting of advanced cancer care were discussed. The patient expressed her understanding and requested to proceed.

Home (HPN) was initiated and advanced slowly because of refeeding risk. Synergistic nutrition support and medical management, in the setting of advanced cancer, allowed for successful initiation of, and ongoing, HPN. Continuous analysis of benefits vs risks/burdens of PN via a transdisciplinary approach was instituted throughout the trajectory of her illness.

The patient was able to spend time at home enjoying and engaging in meaningful conversations with her family and children. The patient attended her children's tennis games and played board games with them. She was able to meet a milestone to be with her family during the holiday season. The patient expressed hope and lived one day at a time, all the while, fully comprehending her cancer diagnosis. The patient stated, “PN has given me more time with my children and family as well as time to pursue nontraditional cancer treatments.” The patient lived for 6 more months.

The institution was consulted, and publishing a single case report of a deceased patient did not require Institional Review Board approval. The institution recommened obtaining consent from the family to publish the case report without identifiers, and this was done.

The case illustrates the opportunity presented by application of the ethical biopsy checklist guided by the core ethical principles of autonomy, beneficence, nonmaleficence, and justice. Ethical issues can be addressed and concerns regarding the complexities of PN at EOL can be resolved (Table 1).

Another aspect of the ethical biopsy is that it provides the necessary armamentarium to the healthcare team to justify financial coverage (insurance) for provision of AANH for patients receiving palliative or hospice care as a function of their moral obligation to the community at large (ethical dimension of T3).^6^

## CONCLUSION

The ethical biopsy is a novel approach for addressing ethical dilemmas and reaching ethically sound and patient‐centered conclusions via a structured framework applying ethical principles. It offers a practical and dignified response to ethical uncertainty and tensions in clinical nutrition and considerations of AANH in EOL care. Rooted in the ethical dimension of T3, it ensures decisions are made via an inclusive, informed, and compassionate dialogue. By instituting an ethical framework, or ethical biopsy, clinicians can more effectively navigate complex decisions in nutrition support and EOL care.

By extending the concept of a biopsy into the ethical realm, clinicians are empowered to uncover the hidden causes of ethical tension and chart a course applying ethical principles that respects both the science and soul of patient care. Additionally, the ethical biopsy can better equip individuals to advocate for financial coverage for AANH during EOL care, if indicated and desired by the patient.

Nota bene: The terms “disciplinary” and “transdisciplinary” referred to in this article are inclusive of the more recently terminology “professional” and “trans(inter)professional.” Subsequent to the initial submission of this article a comprehensive review, published online, provides a more detailed discussion of various elments of the Ethical Biopsy^8^ adding to those in the recently published ASPEN's 4th Adult Nutrittion Support Core Curriculum Chapter (22) "Ethical and Legal Foundations in Nutrition Support.^3^

## AUTHOR CONTRIBUTIONS

Albert Barrocas developed the concept and design of the manuscript; Alison Evans provided the narrative of the example case; and Albert Barrocas and Alison Evans developed Table 1 and drafted the manuscript with critical revisions. All authors critically revised the manuscript, agree to be fully accountable for ensuring the integrity and accuracy of the work, and read and approved the final manuscript.

## CONFLICT OF INTEREST STATEMENT

None declared.
